# Early detection of venous thromboembolism after the initiation of chemotherapy predicts a poor prognosis in patients with unresectable metastatic pancreatic cancer who underwent first-line chemotherapy with gemcitabine plus nab-paclitaxel

**DOI:** 10.1371/journal.pone.0264653

**Published:** 2022-03-01

**Authors:** Takuo Yamai, Kenji Ikezawa, Erika Hiraga, Yasuharu Kawamoto, Takeru Hirao, Sena Higashi, Kazuma Daiku, Shingo Maeda, Yutaro Abe, Makiko Urabe, Yugo Kai, Ryoji Takada, Tasuku Nakabori, Nobuyasu Fukutake, Hiroyuki Uehara, Masashi Fujita, Kazuyoshi Ohkawa

**Affiliations:** 1 Department of Hepatobiliary and Pancreatic Oncology, Osaka International Cancer Institute, Osaka, Japan; 2 Department of Onco-Cardiology, Osaka International Cancer Institute, Osaka, Japan; Chang Gung Memorial Hospital and Chang Gung University, Taoyuan, Taiwan, TAIWAN

## Abstract

**Background:**

Pancreatic cancer is associated with a high thromboembolism risk. We investigated the significance of early venous thromboembolism (VTE) detection in patients with unresectable metastatic pancreatic cancer (UR-MPC) who received first-line chemotherapy with gemcitabine plus nab-paclitaxel (GnP).

**Methods:**

This single-center retrospective study enrolled 174 patients with UR-MPC who underwent GnP as a first-line chemotherapy from April 2017 to March 2020. The early detection of VTE (deep venous thrombosis and pulmonary thromboembolism) was defined as diagnosis by the first follow-up CT scan after the initiation of chemotherapy. We compared the patients with early detection of VTE (VTE (+) group) with the others (VTE (-) group). We examined overall survival (OS), progress free survival (PFS), severe adverse events, and predictors associated with OS using the Cox proportional hazards model.

**Results:**

Early detection of VTE was observed in 17 patients (9.8%). Thirteen patients were diagnosed with VTE at treatment initiation, and four patients were diagnosed after treatment initiation. The median time to diagnosis after treatment initiation was 55 days (range: 31–71 days). Only 3 patients were symptomatic. The VTE (+) group exhibited worse OS and PFS than the VTE (-) group (OS: 259 days vs. 400 days, P < 0.001; PFS: 120 days vs. 162 days, P = 0.008). The frequency of grade 3–4 adverse events was not significantly different. Although the performance status was poorer in the VTE (+) group, VTE was identified as a statistically significant independent predictor for OS in multivariate analyses (HR, 1.87; 95% CI, 1.02–3.44; P = 0.041).

**Conclusions:**

Early VTE detection is a predictor of a poor prognosis in UR-MPC patients who receive GnP as first-line chemotherapy, suggesting that screening VTE for patients with UR-MPC is crucial, even if patients are asymptomatic.

## Introduction

Pancreatic cancer is estimated to be the fourth leading cause of death and generally has a poor prognosis, with a 5-year overall survival (OS) rate of 10% at all stages [1]. Pancreatic cancer is difficult to diagnose at an early stage [2, 3], and more than half of patients with pancreatic cancer are diagnosed at unresectable stages [4, 5]. Chemotherapy has contributed to improvements in the prognosis of patients with unresectable pancreatic cancer [4–6].

Gemcitabine (GEM) plus nab-paclitaxel (GnP) combination chemotherapy is a standard regimen for unresectable pancreatic cancer patients with a good performance status (PS) [7]. In the phase 3 MPACT trial, GnP chemotherapy demonstrated a median OS of 8.6 months, a progression-free survival (PFS) of 4.0 months and an objective response rate (ORR) of 23% for patients with unresectable metastatic pancreatic cancer (UR-MPC) [8]. In the real world, almost all patients eventually develop the disease, and a complete cure is difficult to achieve. Selecting the appropriate patient for GnP chemotherapy is necessary. The CA19-9 [9], the serum albumin level [10], older age [10] and the neutrophil-to-lymphocyte ratio [11] have been reported as predictive and pretreatment markers of GnP chemotherapy for patients with UR-MPC. However, the association between venous thromboembolism (VTE) and the prognosis of patients receiving GnP chemotherapy has not been fully established.

Pancreatic cancer has one of the highest rates of thrombotic complications among cancers [12]. The incidence of VTE is 10–20% among all cancers [13, 14]. Thrombosis is the second leading cause of death among outpatients with cancer [13, 14]; thus, VTE is considered a potentially fatal complication in pancreatic cancer patients. Lee et al. reported that overall, VTE did not affect mortality, and the outcome of symptomatic VTE was worse than that of asymptomatic VTE [15]. By contrast, Chen et al. reported that VTE after the diagnosis of pancreatic ductal adenocarcinoma was associated with significant decreases in PFS and OS [16]. Although VTE is detected throughout the entire treatment period, the early detection of VTE predicts poor survival in UR-MPC patients who received palliative chemotherapy [17]. However, no report has evaluated the relationship between the early detection of VTE and efficacy of a specific regimen for pancreatic patients with a uniform stage. Therefore, this study aimed to clarify the impact of the early detection of VTE on the outcome of GnP chemotherapy as a first-line treatment, particularly in UR-MPC patients.

## Materials and methods

### Study design and patients

We retrospectively collected the clinical data of 174 patients with pathologically diagnosed unresectable metastatic pancreatic adenocarcinoma who were treated with GnP chemotherapy as a first-line regimen at our hospital between April 2017 and March 2020. GnP chemotherapy was administered as follows: 30 min of intravenous infusion of nab-paclitaxel at 125 mg/m^2^ followed by 30 min of intravenous infusion of GEM at 1000 mg/m^2^ on days 1, 8, and 15. One cycle was 28 days. The dosages and schedules of therapeutic agents were adjusted as appropriate according to the patient conditions. For each patient, the data were extracted from medical records. The following clinical parameters were obtained: age, sex, Eastern Cooperative Oncology Group (ECOG) PS, primary tumor location, biliary drainage, laboratory data (levels of white blood cells (WBCs), hemoglobin (Hb), platelets (Plt), D-dimer, and carbohydrate antigen 19–9 (CA19-9)), imaging findings before and during treatment, details of GnP chemotherapy (dosages and schedules of therapeutic agents, treatment response, and toxicities), postGnP therapeutic regimens, and OS time. Tumor responses were assessed according to the Response Evaluation Criteria in Solid Tumors (RECIST) ver. 1.1. Hematological and nonhematological adverse events (AEs) were evaluated according to the Common Terminology Criteria for Adverse Events version 5.0. Progression-free survival (PFS) was calculated from the start date of GnP chemotherapy to the date of the assessment of progressive disease or any cause of death. OS was calculated from the start date of GnP chemotherapy to the date of death. Anemic events were defined as those requiring blood transfusion or endoscopic hemostasis. Follow-up data from patients were censored on March 31, 2021. The study was performed in accordance with the Declaration of Helsinki. Approval was obtained from the Ethics Committee of the Osaka International Cancer Center (18225–4). Informed consent was obtained using the opt-out form on the website.

### Definition of VTE

In this study, VTE included deep venous thrombosis (DVT) of the lower extremities and pulmonary thromboembolism. VTE was diagnosed via contrast-enhanced computed tomography (CT) and/or ultrasonography. The early detection of VTE was defined as a diagnosis of VTE by the first follow-up CT scan after the initiation of chemotherapy. Patients whose VTE was detected early were classified into the VTE (+) group. Patients with symptomatic VTE were defined as those who had symptoms due to VTE at diagnosis.

### Statistical analysis

Categorical variables were described as percentages, and continuous variables were presented as medians and ranges. The patient characteristics, treatment outcomes, and toxicities of chemotherapy were compared between the VTE (+) group and other patients (VTE (-) group) using Fisher’s exact test for categorical variables or the Mann–Whitney U test for continuous variables. The log-rank test was used to compare OS and PFS. Univariate and multivariate analyses were performed to identify significant prognostic factors associated with OS using the Cox proportional hazards model. Hazard ratios (HRs) and 95% confidence intervals (CIs) were calculated. Factors with P values less than 0.10 in univariate analysis were entered into multivariate Cox models. For the P value, the significance level was defined as 0.05. Statistical analyses were performed using JMP Ver. 14.0 (SAS Institute, Cary, NC, USA).

## Results

### Patient characteristics

The characteristics of the 174 patients included in the present study are summarized in **Table 1**. Seventeen patients (9.8%) were classified in the VTE (+) group, and three of them were symptomatic. Thirteen patients were diagnosed with VTE at treatment initiation. Four patients were diagnosed after treatment initiation, and the median time to diagnosis after treatment initiation was 55 days (range: 31–71 days). The median age was similar in both groups (VTE (+), 63 years (range: 45–79 years) vs. VTE (-), 65 years (range: 41–81 years); *P* = 0.947). No significant difference was found in the baseline body mass index between the two groups (VTE (+), 21.6 (range: 17.0–34.2) vs. VTE (-), 20.9 (range: 15.2–33.0); *P* = 0.187). Although the proportion of male patients was lower in the VTE (+) group, the difference did not reach statistical significance (*P* = 0.073). Approximately 40% of tumors in both groups were located in the pancreatic head. The percentage of patients who underwent biliary drainage was not significantly different (41.2% in the VTE (+) group vs. 31.8% in the VTE (-) group; *P* = 0.428). The percentage of patients with PS = 0 was significantly lower in the VTE (+) group (35.3% in the VTE (+) group vs. 63.7% in the VTE (-) group; *P* = 0.034). Although the levels of WBCs, Hb, Plt, and CA19-9 were not significantly different between the two groups, the baseline D-dimer levels were significantly higher in the VTE (+) group (*P* < 0.001). In the four patients diagnosed with VTE after the start of treatment, the d-dimer levels at the time of VTE diagnosis were elevated compared with those at baseline. The median elevated D-dimer level was 2.8 mg/dl (range, 0.1–8.2 mg/dl). In the VTE (+) group, 13 patients were treated with a direct oral anticoagulant (DOAC), and 3 patients were treated with unfractionated heparin from the time of diagnosis. One patient did not receive anticoagulant therapy.

**Table 1:** Patient characteristics in subgroups according to VTE (+)/VTE (–).

	VTE (+)	VTE(–)	p
Number of patients, n	17	157	
Symptomatic VTE, n (%)	3 (17.6%)		
Asymptomatic VTE, n (%)	14 (82.4%)		
Timepoint of VTE diagnosis			
At treatment initiation, n (%)	13 (76.5%)		
After treatment initiation, n (%)	4 (23.5%)		
Median time to diagnosis (range), days	55 (31–71)		
Median age (range), y.o.	63 (45–79)	65 (41–81)	0.947^†^
Sex			0.073^§^
Male, n (%)	5 (29.4%)	85 (54.1%)	
Female, n (%)	12 (70.6%)	72 (45.9%)	
Baseline median body mass index (range)	21.6 (17.0–34.2)	20.9 (15.2–33.0)	0.187^†^
Location			0.958^§^
Head, n (%)	7 (41.1%)	63 (40.1%)	
Body-tail, n (%)	10 (58.9%)	94 (59.9%)	
Performance status			**0.034** ^§^
0	6	100	
1-	11	57	
Bile duct stenting: Yes, n (%)	7 (41.2%)	50 (31.8%)	0.428^§^
Median WBC (range),/μl	6240 (1020–14680)	6750 (730–10970)	0.260^†^
Median Hb (range), g/dl	12.7 (9.3–15.9)	13.3 (7.8–17.2)	0.059^†^
Median Platelet (range), 10^4^/μl	26.9 (6.2–48.0)	23.0 (11.7–56.5)	0.356^†^
Median D-dimer (range), mg/dl	3.5 (0.8–30.2)	1.8 (0.6–16.7)	**<0.001** ^†^
Median CA19-9 (range), mg/dl	9000 (2–100000)	7500 (2–100000)	0.062^†^
Treatment for VTE			
DOAC, n (%)	13 (76.5%)		
Unfractionated heparin, n (%)	3 (17.6%)		
None, n (%)	1 (5.9%)		

†, Mann–Whitney U test

§, Fisher’s exact test.

VTE, venous thromboembolism; WBC, white blood cell; Hb, hemoglobin; CA19-9, carbohydrate antigen 19–9; DOAC, direct oral anticoagulant.

### Treatment outcomes

**Fig 1:**
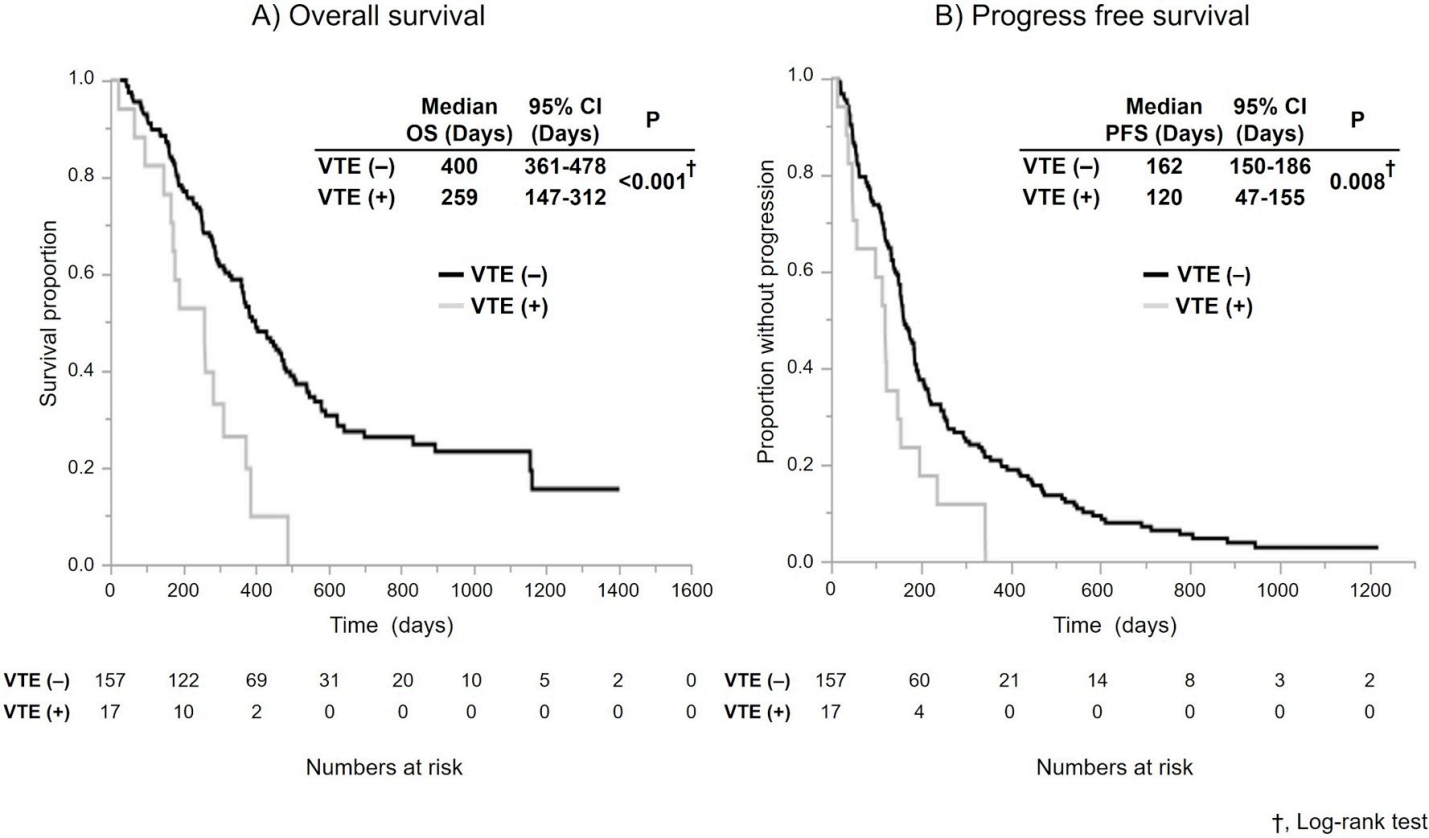
Kaplan–Meier survival curves for (A) OS and (B) PFS. P values were calculated using the log-rank test. OS, overall survival; PFS, progression-free survival; VTE, venous thromboembolism; CI, confidence interval.

The median follow-up period was 350 days (range, 23–1403 days). At the end of the follow-up period, 136 patients (78.1%) died or were censored. The median OS and PFS were significantly shorter in the VTE (+) group than in the VTE (-) group (OS: 259 days [95% CI: 147–312 days] vs. 400 days (95% CI: 361–478 days], *P* < 0.001; PFS: 120 days [95% CI: 47–155 days] vs. 162 days [95% CI: 150–86 days], *P* = 0.008) (**Fig 1**). The tumor response and dose reduction rate in the first course are summarized in **Table 2**. A complete response, a partial response, stable disease, and progressive disease were observed in 0, 5, 6, and 5 patients in the VTE (+) group and 1, 47, 77, and 23 patients in the VTE (-) group, respectively. The objective response rate (ORR) was 29.4% in the VTE (+) group and 30.5% in the VTE (-) group. No significant difference was found in the ORR, disease control rate or dose reduction rate in the first course between the groups.

**Table 2:** Tumor response and dose reduction in the 1st course of therapy.

	VTE (+)	VTE (–)	p
	n = 17	n = 157	
Best Response, n			
Complete response	0	1	
Partial response	5	47	
Stable disease	6	77	
Progress disease	5	23	
Not evaluated	1	8	
Response rate, n (%)	5 (29.4%)	48 (30.5%)	0.414
Disease control rate, n (%)	11 (64.7%)	125 (79.6%)	0.212
Dose reduction in the 1st course, n(%)	13 (76.4%)	103 (65.6%)	0.429

P values were calculated using Fisher’s exact test. VTE, venous thromboembolism.

### Toxicity

No patient died because of AEs due to chemotherapy. Grade 3–4 AEs are summarized in **Table 3**. No significant difference was found in hematological toxicities and nonhematologic toxicities between the groups. Grade 3–4 hematologic toxicities occurred in 10 patients (58.8%) in the VTE (+) group and 102 patients (66.2%) in the VTE (-) group. Grade 3–4 nonhematologic toxicities were observed in six patients (35.3%) in the VTE (+) group and 41 patients (26.6%) in the VTE (-) group. The major grade 3–4 nonhematological toxicities were as follows: peripheral neuropathy (5.9%/6.5%), infection (5.9%/5.2%), constipation (11.8%/3.3%), fatigue (0%/3.9%), and appetite loss (0%/3.9%). Interstitial lung disease was observed in three patients (17.6%) in the VTE (+) group and 16 patients (10.4%) in the VTE (-) group. A total of 40 anemic events occurred during the follow-up period. Although anemic events were more frequent in the VTE (+) group (six patients (35.5%)) than in the VTE (-) group (34 patients (22.1%)), the difference was not statistically significant (*P* = 0.228). No deaths due to hemorrhage or anemia occurred. No cases of ischemic disease occurred during anticoagulation.

**Table 3:** Severe (grade 3–4) adverse events.

	VTE (+)	VTE (–)	p
	n = 17	n = 157	
Death due to an adverse event, n (%)	0 (0.0%)	0 (0.0%)	
Hematologic adverse event (grade 3–4), n (%)	10 (58.8%)	102 (66.2%)	0.604
Neutropenia	4 (23.5%)	68 (44.2%)	0.129
Leukopenia	6 (35.2%)	79 (51.3%)	0.309
Thrombocytopenia	2 (11.8%)	18 (11.7%)	1.000
Anemia	7 (41.2%)	42 (27.3%)	0.256
Nonhematologic adverse event (grade 3–4), n (%)	6 (35.3%)	41 (26.6%)	0.403
(occurring in >3% of patients)			
Peripheral neuropathy	1 (5.9%)	10 (6.5%)	1.000
Infection	1 (5.9%)	8 (5.2%)	1.000
Constipation	2 (11.8%)	5 (3.3%)	0.141
Fatigue	1 (5.9%)	6 (3.9%)	0.519
Appetite loss	1 (5.9%)	6 (3.9%)	0.519
ILD (all grade), n (%)	3 (17.6%)	16 (10.4%)	0.386
Anemic events, n (%)	6 (35.3%)	34 (22.1%)	0.228

P values were calculated using Fisher’s exact test. ILD (all grade), and anemic events. VTE, venous thromboembolism; ILD, interstitial lung disease.

### Post-GnP treatment

Post-GnP treatment is summarized in **Table 4**. Six patients continued GnP chemotherapy at the end of the follow-up period. One patient achieved a complete response and was under observation without treatment. Three patients underwent conversion surgery. Overall, 120 patients received second-line chemotherapy. The percentage of patients who received second-line chemotherapy was lower in the VTE (+) group than in the VTE (-) group (53.0% [9/17] versus 70.7% [111/157]; *P* = 0.071). The second-line therapy regimens in the VTE (+) group were modified FOLFILINOX in two patients, 5-fluorouracil (5-FU) plus nanoliposomal irinotecan (nal-IRI) in one patient, S-1 in five patients, and other regimens in one patient. In the VTE (-) group, modified FOLFILINOX was administered to 46 patients, 5-FU plus nal-IRI to eight patients, S-1 to 48 patients, pembrolizumab to one patient, an investigational agent to one patient, chemoradiation to three patients, and other regimens to four patients. The percentage of patients who chose best supportive care (BSC) was significantly higher in the VTE (+) group (47.0% [8/17] vs. 23.0% [36/157], *P* = 0.034).

**Table 4:** PostGnP treatment at the time of data cutoff.

	VTE (+)	VTE (–)	p
	n = 17	n = 157	
GnP-therapy ongoing, n (%)	0 (0.0%)	6 (3.8%)	
GnP-therapy terminated, n (%)	17 (100.0%)	151 (96.2%)	
Complete response, n (%)	0 (0.0%)	1 (0.6%)	
Conversion Surgery, n (%)	0 (0.0%)	3 (1.9%)	
Second line chemotherapy, n (%)	9 (53.0%)	111 (70.7%)	0.071
mFOLFOLINOX	2 (11.8%)	46 (29.3%)	
5-FU+nal-IRI	1 (5.9%)	8 (5.1%)	
S-1	5 (29.4%)	48 (30.7%)	
Pembrolizumab	0 (0.0%)	1 (0.6%)	
Investigational agent	0 (0.0%)	1 (0.6%)	
Chemoradiotherapy	0 (0.0%)	3 (1.9%)	
Other	1 (5.9%)	4 (2.5%)	
BSC, n (%)	8 (47.0%)	36 (23.0%)	**0.034**

P values were calculated using Fisher’s exact test. FOLFIRINOX, oxaliplatin, irinotecan, 5-FU and leucovorin; nal-IRI, nanoliposomal irinotecan; BSC, best supportive care.

### Factors associated with OS

Finally, we examined the predictive factors associated with OS (**Table 5**). In univariate analysis of OS, four variables were significantly associated with OS: ECOG PS (HR, 2.93; 95% CI, 2.04–4.21; *P* < 0.001), VTE (+) (HR, 6.39; 95% CI, 1.40–29.0; *P* = 0.016), baseline CA19-9 levels (HR, 1.62; 95% CI, 1.13–2.32; *P* = 0.008) and baseline D-dimer levels (HR, 1.84; 95% CI, 1.28–2.64; *P* < 0.001). Multivariate analysis was performed using these four variables. VTE was identified as a statistically significant independent predictor of OS (HR, 1.87; 95% CI, 1.02–3.44; *P* = 0.041), ECOG PS (HR 2.52, 95% CI, 1.73–3.66, *P* < 0.001) and the CA19-9 levels (HR 1.75, 95% CI 1.02–3.44, *P* = 0.003).

**Table 5:** Univariate and multivariate analyses of OS.

	Univariate analysis			Multivariate analysis		
	HR	95% CI	p	HR	95% CI	p
Age (>70 vs. <70, y.o.)	1.19	0.79–1.80	0.389			
Male vs. Female	0.85	0.47–1.52	0.594			
Performance status (1- vs. 0)	2.93	2.04–4.21	**<0.001**	2.52	1.73–3.66	**<0.001**
Tumor location (Head vs. other)	1.17	0.38–1.67	0.388			
VTE (VTE(+) vs. VTE(-))	6.39	1.40–29.0	**0.016**	1.87	1.02–3.44	**0.041**
Bile duct stenting (Y vs. N)	1.16	0.80–1.68	0.409			
Baseline WBC (>9000 vs. ≦9000,/μL)	1.92	0.81–4.59	0.136			
Baseline Hb (<10.0 vs. ≧10.0, g/dL)	1.38	0.60–3.14	0.442			
Baseline Platelet (>25.0 vs. ≦25.0, ×10^4^/μL)	1.04	0.72–1.50	0.802			
Baseline CA19-9 (>1000 vs. ≦1000, U/mL)	1.62	1.13–2.32	**0.008**	1.75	1.02–3.44	**0.003**
Baseline D-dimer (>2.0 vs.≦2.0, μg/mL)	1.84	1.28–2.64	**<0.001**	1.48	0.99–2.20	0.051

Univariate and multivariate analyses of these variables were performed using the Cox proportional hazard regression model. OS, overall survival. OS, overall survival; WBC, white blood cell; Hb, hemoglobin

## Discussion

This study revealed that the early detection of VTE was associated with a poor prognosis in patients with UR-MPC who underwent GnP as a first-line chemotherapy. Although VTE is considered a critical complication in patients with UR-MPC, the impact of early VTE detection remains to be fully elucidated in patients who receive multidrug combination therapy, including GnP. To our best knowledge, this report is the first to identify early VTE detection as a prognostic factor for patients with UR-MPC who receive first-line GnP chemotherapy, which is a standard chemotherapeutic regimen. Based on the results described above, we uncovered the following three crucial findings.

First, the OS and PFS were significantly shorter in the VTE (+) group in this study. The OS and PFS of the VTE (-)and VTE (+) groups were 400 vs. 259 days (OS; *P* < 0.001) and 162 vs. 120 days (PFS; *P* = 0.008), respectively. In a Japanese phase 1/2 study, GnP chemotherapy led to median PFS and OS times of 6.5 months (95% CI, 5.1–8.3) and 13.5 months (95% CI, 10.6—not reached), respectively [18]. Our results showed that the OS and PFS in the VTE (-) group were similar to those in the Japanese phase 1/2 study, and the OS and PFS were both significantly shorter in the VTE (+) group than in the VTE (-) group. Although previous retrospective studies reported no association between VTE and overall survival (OS) in pancreatic cancer patients [19, 20], recent studies have reported that the diagnosis of VTE was associated with a 1.6-fold risk decrease in OS [21]. This difference was presumably due to the improved OS associated with the progression in chemotherapy and supportive care. Additionally, our results revealed more patients with advanced disease in the VTE (+) group. In the VTE (+) group, a high proportion of patients had higher CA19-9 levels and a low percentage of patients had PS = 0. Although the RR, disease control rate and dose reduction in the first course were not significantly different in VTE (+) and VTE (-) groups, the proportion of patients who chose BSC after GnP chemotherapy was significantly higher in the VTE (+) group. Patients in the VTE (+) group likely could not receive second-line chemotherapy because of the worsening of their general condition at the time of disease progression. Our analysis implies that the early diagnosis of VTE is associated with potential pancreatic cancer progression and reduces the probability of post-GnP treatment.

Second, more than 80% of the patients in the VTE (+) group were asymptomatic. In our study, all asymptomatic patients with high D-dimer levels underwent ultrasonography of the lower extremities to exclude DVT, enabling us to detect asymptomatic cases. A systematic review of patients with pancreatic carcinoma reported that the incidence of VTE was 5.0–36.0% [22]. In a Japanese cohort of 107 chemo-naïve patients with pancreatic cancer, 17 (16.5%) were diagnosed with VTE; in particular, only 3 patients were symptomatic [23]. Although VTE is a potentially fatal disease, pancreatic cancer patients with VTE rarely have comorbid symptoms of VTE. In our study, the total incidence rate of VTE (9.7%) was not higher than that in previous reports because of the limited period of diagnosis. Additionally, the high proportion of asymptomatic cases (> 80%) was similar to that of a previously described Japanese cohort [23]. Asymptomatic patients likely could not be diagnosed with VTE because of the measurement of the D-dimer level at diagnosis and follow-up. Asymptomatic VTE is difficult to detect without various assessments. Therefore, not only diagnostic imaging tests, such as CT scans but also useful biomarker assessments are needed to detect asymptomatic VTE. Patients with VTE frequently exhibited elevated D-dimer, fibrin degradation product, and IL-6 levels. Additionally, factor VIII, D-dimers, von Willebrand factor, free tissue factor pathway inhibitors, microvesicle-tissue factor activity and CA 19–9 levels have been reported as important biomarkers to assess VTE risk [24]. Among these biomarkers, D-dimer is particularly useful because of the correlation among pancreatic cancer, coagulation activity and fibrinolysis [23]. Thus, our study showed that monitoring the D-dimer levels at and after treatment initiation may be a highly sensitive method for the early diagnosis of asymptomatic VTE. The early diagnosis of VTE can contribute to the prevention of fatal VTE, as described below. Additionally, although more than 80% of patients were asymptomatic, early VTE detection was a poor prognostic factor for GnP chemotherapy as the first-line regimen.

Third, our results showed that no patient had recurrent VTE or ischemic disease, including brain infarction and myocardial infarction, during anticoagulation, and no difference was found in the frequency of severe anemia events between the groups. Almost all the patients in the VTE (+) group were treated with a direct oral anticoagulant (DOAC) or unfractionated heparin from the time of diagnosis, and all the patients continued to receive thromboprophylaxis unless they had a severe anemia event. Anticoagulation therapy has been recommended to prevent the worsening of VTE and reduce mortality. By contrast, VTE has a high recurrence rate, and anticoagulation is associated with bleeding events, including gastrointestinal bleeding [14, 25]. Recently, a meta-analysis of 1003 pancreatic cancer patients has revealed that anticoagulation therapy significantly reduced the risk of symptomatic VTE without increasing major bleeding [26]. Considering these results, the International Initiative on Thrombosis and Cancer (14) and American Society of Clinical Oncology Clinical Practice [25] guidelines recommend anticoagulation therapy with apixaban or rivaroxaban in cancer outpatients undergoing chemotherapy with a Khorana score ≥ 2, no bleeding risk and no drug-drug interactions (Grade 1B) [14]. Thus, these guidelines indicate that thromboprophylaxis may now be considered in all ambulatory pancreatic cancer patients, given that 2 points are assigned for the primary site being the pancreas in the Khorana score [27]. However, the benefit of anticoagulation therapy in these patients continues to be underrecognized worldwide. The reasons for this are mainly due to the fear of severe bleeding and inherent costs for anticoagulant therapy [28]. Given the short OS of pancreatic cancer mentioned above, no strong evidence exists that anticoagulation therapy has contributed to improving the overall survival of advanced pancreatic cancer patients who receive chemotherapy [16, 29, 30]. Based on these data, anticoagulation therapy effectively prevented ischemic diseases. Taken together, we suggest that the indications and durations for anticoagulation therapy must be adjusted in each patient, and appropriate follow-up evaluations are also required to detect severe anemia due to gastrointestinal bleeding and/or myelosuppression.

This study has limitations. First, this study was a single-center, retrospective analysis. Treatment evaluation was based on the judgment of each attending physician, and secondary evaluations were not performed in this study. Second, the sample size of the study was small. Thus, to clarify the significance of the early detection of VTE, further multicenter, large-scale and prospective studies are required.

In conclusion, early detection of VTE predicts a poor prognosis in patients with UR-MPC who receive first-line GnP chemotherapy. Our investigation indicates that screening for VTE in patients with UR-MPC will be crucial, even if the patients are asymptomatic.
